# Metabolic sovereignty through oxidative hostility: a mechanistic perspective on how cancer engineers stromal dependency via ROS-mediated lysosomal reprogramming

**DOI:** 10.3389/fonc.2026.1876377

**Published:** 2026-06-30

**Authors:** Khalid O. Alfarouk, Saeed Alshahrani

**Affiliations:** 1 Alfarouk Biomedical Research LLC, Valdosta, GA, United States; 2 College of Pharmacy, Jazan University, Jazan, Saudi Arabia

**Keywords:** cancer-associated fibroblasts, fatty acid oxidation, Fenton chemistry, H_2_O_2_, immunotherapy resistance, lysosomal membrane permeabilization, metabolic reprogramming, NOX

## Abstract

Cancer cells orchestrate a profound remodeling of their microenvironment to suppress immune surveillance and create metabolic dependency in surrounding stroma. We propose a mechanistic hypothesis in which this transformation is driven by a coordinated reactive oxygen species (ROS) signaling cascade. Cancer cells generate superoxide (O_2_•^-^) through NADPH oxidase (NOX) upregulation and mitochondrial respiration. Superoxide is rapidly converted to hydrogen peroxide (H_2_O_2_), a stable, membrane-diffusible ROS species that crosses stromal cell membranes via aquaporin channels. Within cancer-associated fibroblasts (CAFs), H_2_O_2_ triggers controlled lysosomal membrane permeabilization (LMP), releasing catalytic iron and initiating iron-catalyzed Fenton chemistry that converts this signal into reactive lipid-peroxidation products, which in turn activate PGC-1α and drives a profound shift in CAF metabolism toward fatty acid oxidation (FAO). Through this cascade, CAFs become predominantly FAO-dependent, producing acetyl-CoA, NADPH, and ATP that fuel tumor growth while simultaneously generating a lactate-enriched, acidic, nutrient-depleted microenvironment hostile to immune function. We present three converging lines of evidence supporting this mechanism and provide four experimentally falsifiable predictions, including a critical iron chelation experiment designed as the crucial mechanistic validation of the cascade. If validated, this framework redefines immunotherapy resistance as a metabolic infrastructure problem—not only an immune cell problem—and predicts that targeting stromal metabolic engineering in combination with checkpoint blockade may circumvent resistance in cold tumors.

## 1 Introduction

Cancer immunotherapy has produced remarkable and durable responses in selected tumor types, yet the majority of solid tumors remain resistant to checkpoint blockade. This resistance is not explained by a simple absence of immune cells—CD8+ T cells, NK cells, and macrophages frequently infiltrate resistant tumors but fail to eliminate cancer cells. Removing the PD-1/PD-L1 brake does not restore their function. The question that demands a mechanistic answer is not why immune cells are absent, but why they are present and yet functionally disabled.

The prevailing framework attributes this dysfunction to cell-intrinsic immune suppression: TGF-β signaling, regulatory T cell accumulation, and MHC-I downregulation on tumor cells. These mechanisms are real and clinically relevant, but they are incomplete. They explain why individual immune cells are silenced but not why the entire immune response fails at the tissue level even after checkpoints are released. We propose that the answer lies in a distinct and complementary axis: the metabolic engineering of the tumor microenvironment (TME) by cancer cells acting through cancer-associated fibroblasts (CAFs).

**Figure 1:**
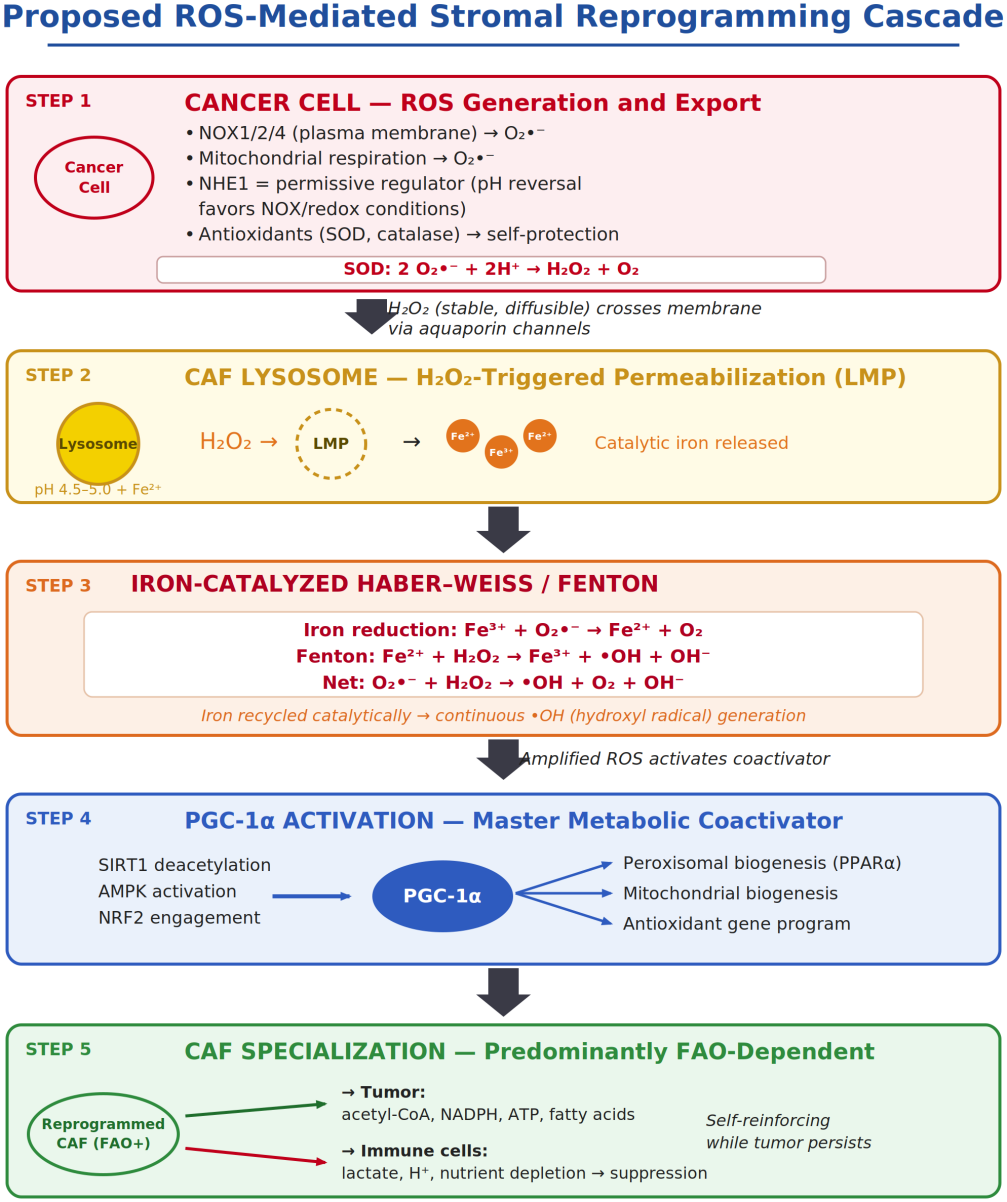
The proposed five-step ROS-mediated cascade by which cancer cells reprogram cancer-associated fibroblasts (CAFs). Cancer cells generate superoxide (O_2_•^-^) through NOX upregulation and mitochondrial electron transport chain activity, while protecting their own redox homeostasis through upregulated antioxidant defenses. Superoxide undergoes rapid dismutation by superoxide dismutase (SOD) to generate hydrogen peroxide (H_2_O_2_), a membrane-permeable, stable ROS species that crosses cell membranes via aquaporin channels. Within CAFs, H_2_O_2_ initiates controlled lysosomal membrane permeabilization (LMP), releasing catalytic iron into the cytoplasm. Free iron drives Fenton chemistry, converting H_2_O_2_ into the highly reactive hydroxyl radical (•OH) via the iron-catalyzed Haber–Weiss reaction. Because •OH acts only locally, the signal is relayed by diffusible lipid-peroxidation products (e.g., 4-HNE), which engage KEAP1/NRF2 and converge with energetic-stress signaling on PGC-1α to drive peroxisomal and mitochondrial biogenesis. The resulting reprogrammed CAF becomes predominantly FAO-dependent, producing metabolic substrates fueling the tumor while contributing lactate and protons to the immunosuppressive microenvironment. Iron chelation at Step 3 is the proposed critical mechanistic test of the cascade.

We hypothesize that cancer cells actively reprogram CAFs through a specific, ordered cascade initiated by ROS generation and export, amplified by iron-catalyzed Fenton chemistry, and sustained by PGC-1α-driven metabolic remodeling (**Figure 1**). The outcome is a stable stromal metabolic infrastructure that produces lactate, consumes nutrients, and maintains an immunosuppressive chemical environment—a metabolic desert in which immune cells cannot function regardless of their activation state. This Perspective integrates published evidence from cancer metabolism, ROS signaling, lysosomal biology, and tumor immunology to present this cascade as a testable mechanistic hypothesis.

## 2 The tumor microenvironment as organized metabolic warfare

### 2.1 Metabolic abnormalities and their immune consequences

The TME is characterized by metabolic conditions that would be pathological in normal tissue: intratumoral pH of 6.2–6.9 (versus 7.3–7.4 in normal tissue), lactate concentrations of 40 mM or higher, severe hypoxia, glucose depletion, and amino acid scarcity (1–3). These are not passive byproducts of rapid tumor growth. They are organized features maintained by cancer-stromal crosstalk, and each has direct consequences for immune cell function.

High extracellular lactate suppresses T cell receptor signaling and effector cytokine production, stabilizes HIF-1α in dendritic cells and T cells to drive regulatory phenotypes, and activates GPR65/GPR68 proton-sensing receptors to promote Treg differentiation (4–6). Low pH directly impairs NK cell cytotoxicity and T cell effector function (7, 8). Hypoxia shifts tumor-associated macrophages toward immunosuppressive M2-like phenotypes (9, 10). Nutrient scarcity prevents the rapid biosynthetic expansion required for effective immune effector responses (11, 12). Checkpoint inhibitors release the PD-1/PD-L1 brake but cannot restore immune function in a microenvironment engineered to disable it metabolically.

### 2.2 Cancer-associated fibroblasts as metabolic architects

CAFs are the principal architects of the TME metabolic landscape. Unlike normal fibroblasts, CAFs are activated stromal cells reprogrammed to sustain tumor growth. Functionally distinct subsets—iCAFs, myCAFs, and apCAFs—share a common theme: they produce what the tumor requires. Lactate, fatty acids, suppressive cytokines, and extracellular matrix components are all CAF products that create the conditions for tumor expansion and immune evasion (13, 14).

Understanding how the tumor reprograms CAFs is therefore central to understanding TME construction. We propose that the initiating signal is ROS-mediated lysosomal stress, amplified by Fenton chemistry, and sustained through PGC-1α-driven metabolic remodeling that shifts CAFs toward a predominantly FAO-dependent phenotype. This is not a consequence of passive exposure—it is a directed reprogramming strategy.

## 3 The proposed five-step mechanistic cascade

The following cascade is supported by published evidence from five independent biological domains and is summarized schematically in **Figure 1**. The complete integrated chain remains to be validated experimentally as a unified mechanism; each individual step is supported by existing literature.

### 3.1 Step 1 — cancer cells generate and export ROS as a paracrine signal

Cancer cells are net producers of reactive oxygen species (ROS), primarily through two mechanisms: NADPH oxidase (NOX1, NOX2, NOX4) upregulation at the plasma membrane, and mitochondrial electron transport chain (ETC) activity (15). Unlike normal cells, cancer cells demonstrate remarkable metabolic plasticity—aerobic glycolysis is a defining feature, but mitochondrial oxidative phosphorylation may be increased, maintained, or reduced depending on tumor type, oxygen availability, oncogenic signaling, and microenvironmental conditions (16–18).

Superoxide (O_2_•^-^) generated by NOX and mitochondria is rapidly dismutated by superoxide dismutase (SOD): 2 O_2_•^-^ + 2H^+^ → H_2_O_2_ + O_2_ (19). This conversion is mechanistically crucial: superoxide has a half-life of microseconds and is membrane-impermeant (does not cross cell membranes), whereas hydrogen peroxide (H_2_O_2_) is stable (half-life of seconds to minutes in cellular contexts) and membrane-diffusible. H_2_O_2_ crosses stromal cell membranes efficiently through aquaporin water channels, positioning it as the actual intercellular ROS signal.

Paradoxically, cancer cells simultaneously upregulate antioxidant defenses—superoxide dismutase, catalase, glutathione peroxidase—to levels that protect their own redox homeostasis. This creates an asymmetry: intracellular ROS is neutralized in cancer cells; extracellular H_2_O_2_ accumulates in the stroma. This asymmetry is mechanistically consistent with a directed ROS export strategy. Critically, NHE1 (Na+/H+ exchanger 1) acts as a permissive regulator of this process: NHE1-driven intracellular alkalinization creates conditions favoring NOX and mitochondrial ROS generation, while extracellular acidification enhances Fenton reaction kinetics (see Step 3) (20–22). Thus, NHE1 facilitates ROS-mediated stromal communication through redox signaling, rather than directly exporting ROS itself (23, 24).

### 3.2 Step 2 — H_2_O_2_ triggers controlled lysosomal membrane permeabilization in CAFs

Lysosomes are among the most oxidatively vulnerable organelles. Their membranes are enriched in polyunsaturated lipids susceptible to ROS-driven lipid peroxidation (19, 25). Their luminal environment (pH 4.5–5.0) contains catalytic iron liberated from degraded heme proteins and iron-sulfur clusters. When exported H_2_O_2_ reaches CAF lysosomes, it initiates lipid peroxidation of the lysosomal membrane, destabilizing its integrity (25, 26).

At moderate—rather than lethal—oxidative doses, this permeabilization is controlled and sublethal, consistent with the concept of lysosomal membrane permeabilization (LMP) as a stress-response signal rather than a cell death trigger. The lysosomal damage response (LDR) activates adaptive transcriptional programs that remodel metabolism toward survival (25, 27). This is the critical mechanistic pivot: lysosomal rupture as a paracrine metabolic signal rather than a cytotoxic event.

### 3.3 Step 3 — fenton chemistry converts the signal into a localized lipid-peroxidation relay (iron-catalyzed Haber–Weiss reaction)

LMP releases sequestered lysosomal iron into the cytoplasm. In the presence of hydrogen peroxide, this free iron catalyzes the iron-catalyzed Haber–Weiss reaction, which operates via two coupled steps (26, 28):


$$\text{Iron reduction: }{\text{Fe}}^{3+}\;+\;{\text{O}}_{2}^{\cdot^{-}}\;\to \;{\text{Fe}}^{2+}\;+\;{\text{O}}_{2}$$

$$\text{Fenton: }{\text{Fe}}^{2+}\;+\;{\text{H}}_{2}{\text{O}}_{2}\;\to \;{\text{Fe}}^{3+}\;+\;\cdot \text{OH }+\;{\text{OH}}^{-}$$

$$\text{Net Haber-Weiss: }{\text{O}}_{2}{•}^{-}\;+\;{\text{H}}_{2}{\text{O}}_{2}\;\to \;\cdot \text{OH }+\;{\text{O}}_{2}\;+\;{\text{OH}}^{-}$$

The hydroxyl radical (•OH) is the most reactive oxygen species in biology, reacting at near diffusion-limited rates with the nearest biological molecule within nanometers of its production (29, 30). Its mean free path is therefore too short for •OH to act as a long-range intracellular messenger. Rather than amplifying the signal in quantity—the net yield of •OH is stoichiometrically bounded by the available H_2_O_2_ and superoxide—the iron-catalyzed Haber–Weiss reaction converts a mild, diffusible oxidant (H_2_O_2_) into a far more reactive species that executes localized damage; the iron itself is recycled, as superoxide reduces Fe³^+^ back to Fe²^+^, so the reaction is catalytic with respect to iron. We therefore propose that the spatial signal is propagated not by •OH itself but by a lipid-peroxidation relay: •OH attacks polyunsaturated fatty acids in the perilysosomal membrane, generating stable, diffusible reactive electrophilic species—principally 4-hydroxy-2-nonenal (4-HNE)—that escape the immediate microenvironment and reach downstream metabolic sensors (31).

### 3.4 Step 4 — amplified ROS activates PGC-1α and drives metabolic remodeling

The diffusible electrophile 4-HNE links the localized oxidative event to the transcriptional response: it forms covalent adducts on reactive cysteine residues of KEAP1, releasing NRF2 to drive antioxidant gene expression, and acts as a recognized second messenger of oxidative stress (31). NRF2 activation, together with energetic-stress signaling (SIRT1/AMPK), converges on PGC-1α—the master transcriptional coactivator of oxidative metabolism (32–34). Crucially, this adaptive, pro-survival output depends on a sublethal oxidative dose: low-level lipid peroxidation and 4-HNE favor NRF2-mediated adaptation, whereas an overwhelming burst drives ferroptotic cell death rather than reprogramming—defining a dose- and kinetics-dependent boundary the model must respect (31). We further note that the effect of 4-HNE on AMPK is concentration- and context-dependent and not invariably activating; the precise weighting of these inputs in CAFs remains to be determined. Once activated, PGC-1α coordinates a metabolic remodeling program.

Of particular mechanistic importance, PGC-1α drives peroxisomal biogenesis (35); notably, this peroxisomal remodeling has been reported to proceed through a PPARα-independent mechanism. Peroxisomes serve dual functions: they house catalase and peroxidase enzymes that increase antioxidant capacity, and they are primary sites of long-chain fatty acid beta-oxidation. CAF peroxisomal expansion therefore simultaneously increases resistance to ongoing ROS exposure and commits the cell to FAO as a primary metabolic strategy. Concurrent PGC-1α-driven mitochondrial biogenesis further amplifies oxidative phosphorylation capacity and FAO throughput (36, 37).

### 3.5 Step 5 — metabolic specialization: CAFs become predominantly FAO-dependent

With expanded peroxisomal and mitochondrial networks, the reprogrammed CAF becomes predominantly FAO-dependent, generating acetyl-CoA, NADPH, and ATP as primary metabolic products. These products serve the tumor directly through metabolic crosstalk and sustain lactate-mediated immune suppression. Importantly, CAF populations are heterogeneous—some CAFs retain alternative or mixed metabolic profiles (iCAFs, for example, may maintain glycolytic capacity)—but the PGC-1α-activated subset exhibits a pronounced FAO specialization (13, 14). The fatty acids fueling this program are unlikely to be generated *de novo* within a nutrient-poor stroma; candidate sources include lipids released by neighboring cancer cells, adipocyte lipolysis at the tumor margin, and autophagy-derived fatty acids—an upstream fuel supply the model identifies as an open question.

We hypothesize that this phenotype is self-reinforcing for as long as the initiating conditions—cancer-cell ROS export, low pH, nutrient limitation—persist. Whether the reprogrammed state is genuinely stable or reversible upon withdrawal of these signals has not been established by longitudinal study and remains an open question; the directionality of the proposed relationship, not its permanence, is the claim advanced here.

## 4 Converging evidence: three independent lines

The proposed cascade draws support from three independent lines of evidence that converge from distinct biological domains.

### 4.1 Line 1 — ROS consistently drives PGC-1α-mediated metabolic rewiring

Across cell types including cardiomyocytes, neurons, skeletal muscle cells, and macrophages, oxidative stress activates PGC-1α–mediated programs that increase mitochondrial and peroxisomal biogenesis and shift metabolism toward FAO (33, 34). This is a well-established cellular adaptive response, not a cancer-specific phenomenon. Its operation in CAFs under sustained ROS exposure is mechanistically coherent with known biology.

### 4.2 Line 2 — CAFs exhibit FAO-enriched phenotypes *in vivo*

Published studies across multiple cancer types—hepatocellular carcinoma, colon cancer, breast cancer—demonstrate that tumor-adjacent CAFs upregulate FAO-related genes including CPT1A, ACADM, and HADHA; increase mitochondrial number and membrane potential; and produce acetyl-CoA and other metabolic substrates that support tumor proliferation (13, 14). The FAO-enriched phenotype is not an experimental artifact—it has been observed in human tumor specimens. However, CAF metabolic heterogeneity is acknowledged: not all CAFs exhibit identical FAO profiles, and iCAF, myCAF, and apCAF subsets show distinct metabolic signatures.

### 4.3 Line 3 — lysosomes function as ROS-sensitive metabolic sensors

The view of lysosomes as purely degradative organelles has been substantially revised. Lysosomal rupture is increasingly understood as a regulated signaling event that activates the lysosomal damage response, a transcriptional program including antioxidant and metabolic gene induction (25, 27, 38). This positions LMP as a mechanism by which extracellular ROS signals can be transduced into intracellular metabolic reprogramming—a critical mechanistic link in the proposed cascade.

## 5 An ecological perspective: cancer as stromal parasite

Mycobacterium tuberculosis (Mtb) survives inside macrophages—cells evolutionarily designed to destroy it—by manipulating the phagosomal environment. Mtb recruits host protein coronin 1 to prevent phagosome-lysosome fusion, disabling the macrophage’s primary degradative machinery (39–41). The macrophage is commandeered: its cellular infrastructure serves the pathogen rather than its host.

Cancer’s strategy is mechanistically analogous at the intercellular level. Rather than preventing lysosomal fusion (as Mtb does intracellularly), cancer cells trigger controlled lysosomal rupture in adjacent CAFs to initiate metabolic reprogramming. The result is equivalent: the stromal cell is commandeered to serve the invader’s metabolic needs. This ecological framing is more than analogy—it reflects a convergent survival strategy in which exploiting the host cell’s adaptive stress responses is the mechanism of parasitism. Successful tumors, like successful intracellular pathogens, may require metabolic hijacking of host cells as a core survival strategy (42).

## 6 Implications for immunotherapy resistance

The clinical paradox described in Section 1—immune infiltration without immune function—finds a mechanistic explanation in this framework. By the time immune cells arrive at the tumor, the stromal microenvironment has already been metabolically engineered. Reprogrammed CAFs produce lactate that suppresses T cell metabolic fitness, acidify the environment through proton export, deplete glucose and amino acids required for rapid immune cell proliferation, and secrete cytokines including TGF-β and IL-10 that reinforce regulatory immune phenotypes (4, 5, 9).

Checkpoint inhibitors remove the PD-1/PD-L1 signal that inhibits T cell activation. They do not remove the metabolic barriers imposed by stromal engineering. This distinction may explain a consistent clinical observation: checkpoint blockade is most effective in immunologically ‘hot’ tumors with a pre-existing, activated immune infiltrate, and least effective in ‘cold’ tumors characterized by immune exclusion or dysfunction. Cold tumors may be metabolically cold—their stroma has been engineered into a metabolic desert before immune cells can establish functional residence.

This reframing has direct therapeutic implications. If stromal metabolic engineering is a co-driver of immunotherapy resistance, then targeting it alongside checkpoint blockade may be necessary for durable responses in cold tumors. The cascade identifies four specific molecular targets: NOX (Step 1), lysosomal stability (Step 2), iron bioavailability and Fenton chemistry (Step 3), and FAO (Step 5).

A fifth target emerges from the same framework: tumor-associated macrophage (TAM) polarization state. TAMs in the established TME are predominantly M2-polarized—a phenotype classically described as anti-inflammatory and associated with tissue repair, angiogenesis, and immune resolution. This apparent paradox deserves emphasis: although M2 macrophages suppress acute inflammation, they simultaneously create a profoundly immunosuppressive niche that enables tumor immune evasion. M2 TAMs secrete IL-10 and TGF-β, express PD-L1 to engage the PD-1 checkpoint on T cells, and deplete L-arginine through arginase-1 activity—collectively rendering tumor-infiltrating T cells metabolically starved and functionally exhausted. In this context, immune infiltration without immune function is not a failure of T cell recruitment but a consequence of M2-mediated T cell paralysis *in situ*. The metabolic hostility imposed by reprogrammed CAFs—lactate accumulation, pericellular acidification, and nutrient depletion—further reinforces this M2-dominant state by suppressing the pro-inflammatory signaling required for macrophage M1 conversion. By contrast, M1-polarized macrophages execute direct tumoricidal functions: they generate ROS and nitric oxide (NO) as cytotoxic effectors, secrete TNF-α to induce cancer cell apoptosis, and present tumor antigens to activate cytotoxic T lymphocytes via IL-12 secretion. The critical distinction is therefore not between inflammatory and anti-inflammatory macrophages per se, but between a polarization state that kills cancer cells and one that protects them. We propose that targeted ROS elevation—sufficient to drive M2-to-M1 reprogramming in TAMs while remaining below the threshold that promotes CAF-mediated stromal support—represents a fifth mechanistic axis for restoring immune competence in cold tumors. This dose-dependent duality of ROS as both a stromal-engineering signal (at chronic, low levels) and a potential macrophage-reprogramming stimulus (at acute, directed levels) identifies TAM polarization as an integrative node linking the metabolic and immune dimensions of the TME.

## 7 Testable predictions and experimental framework

The proposed cascade generates four experimentally falsifiable predictions. Each can be tested in standard cancer cell biology systems using cancer cell-CAF co-culture models.

### 7.1 Prediction 1 — NOX and SOD inhibition attenuate CAF reprogramming

If NOX-derived superoxide and SOD-mediated H_2_O_2_ generation are central to the directional ROS export, NOX inhibitors (VAS2870, diphenyleneiodonium) and SOD inhibitors/mimetics (DETC, polyethylene glycol-conjugated SOD inhibitors) should reduce the extracellular H_2_O_2_ dose reaching co-cultured CAFs and attenuate downstream reprogramming. Outcome measures: extracellular H_2_O_2_ quantification using genetically encoded HyPer sensors or Amplex Red (DCF-DA is not H_2_O_2_-specific and should be avoided), lysosomal integrity assays (acridine orange or Galectin-3 puncta), PGC-1α protein levels and transcriptional targets, CPT1A expression and oxygen consumption in CAFs, and metabolic flux by 13C-labeled fatty acid tracing.

### 7.2 Prediction 2 — iron chelation blocks the cascade (critical mechanistic test with controls)

This is the critical mechanistic test. If Fenton chemistry and the iron-catalyzed Haber–Weiss reaction are the essential ROS amplification mechanisms between LMP and PGC-1α activation, iron chelation should prevent •OH generation and block PGC-1α activation even when lysosomal rupture is confirmed.

Experimental design: Use both membrane-impermeant (deferoxamine, DFO) and membrane-permeant (deferiprone, SIH) iron chelators. DFO is membrane-impermeant but stabilizes HIF-1α by inhibiting prolyl hydroxylase (PHD), which requires Fe²^+^. This HIF-1α stabilization is a confound for interpreting the mechanism. Deferiprone and other membrane-permeant chelators have differential HIF effects and allow dissociation of iron-Fenton effects from HIF effects.

Critical controls: (1) Pair iron chelation with HIF-1α genetic knockdown or use of PHD stabilizers to isolate Fenton-specific effects from HIF-mediated metabolic effects. (2) Directly detect hydroxyl radical (•OH) by electron spin resonance (ESR/EPR) spin-trapping with DMPO or DEPMPO—the gold standard for Fenton-derived •OH—since common fluorescent probes (DHR123, DCF-DA) are not •OH-specific; complement this with localized lipid-peroxidation read-out by C11-BODIPY ratiometric imaging in the perilysosomal compartment, rather than inferring from downstream PGC-1α activation. (3) Compare outcomes with and without HIF-1α stabilization to show the independence of iron-Fenton effects.

Expected outcome: Iron chelation prevents •OH generation (direct measurement) and PGC-1α activation without preventing LMP. Failure of this prediction—PGC-1α activation persisting despite iron chelation and suppressed •OH generation—would challenge the central role of Fenton chemistry and indicate an alternative amplification pathway.

### 7.3 Prediction 3 — CPT1A inhibition disrupts tumor-supporting crosstalk

CPT1A is the rate-limiting transporter for mitochondrial long-chain fatty acid entry and a direct transcriptional target of PGC-1α/PPARα signaling. CPT1A inhibition with etomoxir or ST1326 should reduce CAF FAO flux and impair production of tumor-supporting metabolites. Outcome measures: CAF oxygen consumption rate by Seahorse assay, acetyl-CoA and NADPH production by metabolomics, and tumor cell proliferation in conditioned medium from treated CAFs. Important caveat: etomoxir exhibits concentration-dependent inhibition of mitochondrial Complex I independent of CPT1A at high doses; dose-titration and genetic validation (CPT1A knockdown) are essential for clean interpretation.

### 7.4 Prediction 4 — combination metabolic targeting synergizes with checkpoint blockade

In syngeneic, stroma-dense tumor models (MC38, CT26, or 4T1), NOX or FAO inhibition alone should produce modest tumor growth delays, while anti-PD-1 monotherapy produces only partial responses. Because effector and memory T cells themselves depend on FAO, concurrent global FAO inhibition risks impairing the very immune cells the strategy aims to liberate; we therefore predict that a sequenced regimen—stromal metabolic remodeling first, followed by checkpoint blockade—will outperform concurrent dosing. The combination should yield superior tumor control with increased tumor-infiltrating lymphocyte (TIL) density and effector function (IFN-γ, granzyme B), reduced intratumoral lactate with restored pH (assessable by microdialysis), and a shift from immune-excluded to parenchyma-infiltrated architecture on multiplexed imaging. This prediction tests both the translational relevance of the cascade and the importance of dosing sequence.

### 7.5 Minimal validation framework

Three sequential experiments constitute a minimal evidence base for the core hypothesis: (1) Confirm directional H_2_O_2_ transfer from cancer cells to CAFs in co-culture using genetically encoded HyPer sensors and direct H_2_O_2_ measurement; (2) Demonstrate that co-culture-derived H_2_O_2_ causes CAF lysosomal permeabilization and cytoplasmic iron accumulation, and that H_2_O_2_-dependent •OH generation occurs via Fenton chemistry (verified by direct •OH measurement); (3) Show that this ROS burden activates PGC-1α and FAO gene expression, and that iron chelation with appropriate controls (addressing HIF-1α confound) blocks •OH generation and PGC-1α activation. Positive outcomes across these three experiments would validate the initiating steps of the cascade and justify full *in vivo* investigation.

## 8 Limitations and experimental gaps

The following limitations constrain the current proposal and define the experimental agenda for testing it.

### 8.1 Biological heterogeneity

CAF heterogeneity presents a significant biological caveat. iCAFs, myCAFs, and apCAFs have distinct transcriptional programs and metabolic states. Whether all subsets respond equivalently to ROS-mediated lysosomal stress, or whether reprogramming is subset-specific, is unknown. Single-cell metabolomic and transcriptomic profiling of CAFs in ROS-exposed co-cultures is needed to resolve this.

### 8.2 Kinetics and ROS dose thresholds

The kinetics and ROS dose threshold of the cascade have not been established. The transition from controlled LMP to cellular protection to PGC-1α-mediated metabolic remodeling likely depends on the magnitude and duration of the ROS signal. Whether the cancer cell-derived ROS dose is sufficient *in vivo* to trigger this cascade independently of other TME signals remains to be determined.

### 8.3 Multi-cellular complexity

The TME contains endothelial cells, myeloid cells, and tissue-resident immune cells that also respond to oxidative stress and metabolic signals. The proposed cascade may operate in concert with, or be modified by, signals from these additional cell types.

### 8.4 Species and tumor-type specificity

Species specificity and tumor-type generalizability have not been established. The mechanism may operate differentially across mouse and human systems and across tumor types with different stromal compositions. Systematic evaluation across multiple cancer types and model systems is required.

### 8.5 Off-target effects of chemical inhibitors

Pharmacological tools frequently exhibit off-target and concentration-dependent effects. Etomoxir inhibits mitochondrial Complex I independent of CPT1A at high concentrations; cariporide and amiloride analogs affect targets beyond NHE1; deferoxamine stabilizes HIF-1α through PHD inhibition, complicating interpretation of iron-specific effects. All pharmacological predictions should be validated orthogonally using genetic approaches (siRNA, shRNA, CRISPR-Cas9) combined with dose-titration and specific biochemical readouts (direct •OH measurement, metabolic flux analysis) to establish clean mechanism.

### 8.6 Requirement for integrated experimental validation

Most importantly, the complete integrated cascade—from cancer cell ROS export through CAF metabolic lockdown—has not been demonstrated in a unified experimental system. Each step is supported by independent literature, but the proposed chain of events remains to be validated as a connected, causal mechanism.

## 9 Therapeutic implications

If validated, the proposed cascade identifies several actionable intervention points. NOX inhibitors (VAS2870, GKT136901) are in clinical development and could reduce the directional ROS signal from cancer cells to stroma. Lysosomal-stabilizing agents (e.g., chloroquine derivatives, sphingosine) might interrupt LMP and prevent cascade initiation (43, 44). Iron chelators (deferoxamine, deferasirox) could block Fenton amplification, with the caveat that HIF-1α confounds must be controlled.

Most translationally compelling is the combination of FAO inhibition with checkpoint immunotherapy. CPT1A inhibitors would block stromal metabolic dependency while checkpoint blockade restores immune activation. The combination would simultaneously dismantle the metabolic infrastructure of the immunosuppressive microenvironment and release the immune brake. This dual targeting addresses both the cause (stromal engineering) and the consequence (immune suppression) of TME construction.

Biomarker development is an integral part of this therapeutic strategy. Tumors with high stromal FAO activity—identifiable by metabolomic profiling or CPT1A immunohistochemistry—may define the patient population most likely to benefit from metabolic combination approaches. The framework thus provides not only therapeutic targets but a rational basis for patient stratification in future clinical trials.

## 10 Conclusion

We have proposed a five-step mechanistic cascade linking cancer cell ROS export to CAF metabolic reprogramming through H_2_O_2_-mediated lysosomal biology and iron-catalyzed Fenton chemistry. Three converging lines of evidence support the plausibility of this mechanism; four falsifiable predictions with detailed experimental designs provide a clear roadmap for testing it. The cascade reframes immunotherapy resistance—at least in a subset of cold tumors—as a metabolic infrastructure problem created by stromal engineering, not merely a failure of immune cell activation.

The critical iron chelation experiment (Prediction 2) with appropriate controls to dissociate Fenton-specific effects from HIF-mediated confounds provides a direct test of the cascade’s central amplification mechanism and can be conducted in standard co-culture systems. If confirmed, this single result would justify a systematic investigation of the complete cascade in preclinical models and, ultimately, in clinical trials combining metabolic and immune targeting strategies.

The tumor’s engineering of stromal dependency is not an inevitable feature of cancer. It is a tractable mechanism with identifiable molecular steps, testable predictions, and therapeutic vulnerabilities. Understanding it may be essential to overcoming immunotherapy resistance in the tumors that currently benefit least from the checkpoint era.
